# Human Health and Ecological Risk Assessment of Heavy Metal Contamination in the Tigris River (Mosul, Iraq): A Spatial–Temporal Analysis Using CCME-WQI and HPI

**DOI:** 10.3390/toxics14060463

**Published:** 2026-05-25

**Authors:** Zena Altahaan, Daniel Dobslaw

**Affiliations:** 1 Institute of Sanitary Engineering, Water Quality and Solid Waste Management, University of Stuttgart, D-70569 Stuttgart, Germany; 2 Institute of Spatial and Regional Planning, University of Stuttgart, D-70569 Stuttgart, Germany; daniel.dobslaw@iswa.uni-stuttgart.de

**Keywords:** water quality assessment, heavy metal pollution, post-conflict environment, human health risk, CCME-WQI, HPI, Mosul, Iraq

## Abstract

River water quality assessments are commonly conducted under conventional anthropogenic pressures; however, the long-term environmental impacts of armed conflicts remain insufficiently understood. This study addresses this gap by evaluating the persistence of war-related heavy metal contamination and its associated human health risks in the Tigris River, Mosul, a post-conflict urban system. The results revealed that Cd, Pb, Cr, and Ni concentrations exceeded WHO guideline values across most sites, while Zn remained within acceptable limits. The highest contamination levels were observed in the central urban zone (Zone 3), which was directly affected by military activities. Hazard quotient (HQ) values for Cd and Pb exceeded the safe threshold (HQ > 1) at all sites, identifying them as dominant contributors to toxicity. The cumulative hazard index (HI) reached extremely high levels (>300 in 2022 and >200 in 2023), indicating severe non-carcinogenic health risks despite a slight temporal improvement. Spatially, contamination increased from upstream to downstream, with midstream and downstream areas acting as critical hotspots. Temporally, although pollutant levels declined in 2023, they remained significantly above safe limits, demonstrating limited natural recovery. Overall, the findings provide clear evidence of the long-term persistence of conflict-related contamination and its sustained risks to human health. This study highlights the need for targeted remediation strategies and offers a transferable framework for assessing water quality in conflict-affected river systems.:

## 1. Introduction

River water quality is a critical determinant of ecosystem health and human well-being, directly impacting drinking water supplies, agricultural irrigation, industrial processes, and recreational activities worldwide [1]. Heavy metals, a significant class of environmental contaminants, pose a particular threat to aquatic ecosystems due to their persistence, bioaccumulation, and potential toxicity to living organisms [2,3,4]. These pollutants can enter river systems through various anthropogenic activities, including industrial discharges, agricultural runoff, and particularly, uncontrolled waste and residues from conflict zones [5,6,7,8]. The comprehensive assessment of heavy metal contamination in rivers is therefore paramount for environmental management and public health, especially in vulnerable regions [9].

While numerous studies investigated heavy metal pollution in rivers under conventional anthropogenic pressures, such as urbanisation and industrialisation, there remains a significant knowledge gap concerning the long-term and specific impacts of armed conflicts on river water quality, particularly regarding heavy metal dynamics in post-conflict environments [10]. War-related activities, including infrastructure damage, the release of munitions constituents, and the disruption of waste management systems, can introduce a unique and often severe spectrum of pollutants into water bodies, differing from typical industrial or agricultural sources [11,12]. The novelty of this study lies in its focused investigation of the Tigris River’s water quality in Mosul, a city profoundly affected by recent conflict, providing crucial insights into the specific nature and extent of heavy metal pollution in such a unique and understudied context.

The Tigris River, a vital water source for Iraq, historically faced various anthropogenic pressures [13]. However, the recent conflict in Mosul and its surrounding areas introduced an unprecedented level of environmental stress, with potential implications for water quality, particularly the accumulation of heavy metals from munitions, damaged infrastructure, and displaced waste [14]. Understanding the post-conflict state of this crucial water body is not merely an academic exercise, but a critical step towards safeguarding public health and facilitating environmental recovery in affected regions [15]. Previous assessments in similar post-conflict areas indicated complex and persistent contamination patterns, necessitating localised and comprehensive investigations to inform effective remediation strategies [16].

In recent years, there has been a growing body of research highlighting the environmental impacts of armed conflicts on water systems across various regions, although such studies remain relatively limited. In Syria, for instance, significant water quality deterioration has been documented, with several studies reporting that concentrations of heavy metals such as Pb and Cd exceeded international drinking water standards due to the destruction of infrastructure and lack of treatment facilities [17]. In Lebanon, research identified organic and heavy metal pollution linked to the legacy of prolonged civil conflict, with contamination levels in some locations exceeding permissible limits for domestic use [18]. Similarly, in the Balkans, the aftermath of aerial bombardment in the 1990s led to the accumulation of heavy metals in surface waters, particularly Pb and Zn, often surpassing environmental quality standards [19]. In Afghanistan, both surface and groundwater have shown signs of degradation due to military activity and the absence of proper sanitation services, with elevated turbidity, nitrate, and heavy metal concentrations exceeding WHO guideline values in several cases [20].

Previous studies highlighted the distinct characteristics of conflict-impacted water systems compared to typical urban–industrial rivers. For example, Schillinger et al. emphasised how armed conflicts disrupt ecological integrity and increase the vulnerability of water bodies to contamination due to lack of regulation and monitoring [21]. Furthermore, recent work by Forester demonstrated that war-related disturbances can enhance the mobility of metals such as lead and cadmium through changes in sediment dynamics and redox conditions, increasing the risk of bioavailability and ecological toxicity [22]. These studies provide an important framework for understanding the environmental dimensions of warfare, yet Iraq remains significantly underrepresented in the literature despite experiencing repeated conflicts. This underscores the importance of the current study, which aims to address this gap by providing a detailed assessment of post-war water quality in the Tigris River near Mosul using internationally recognised indices and quantitative analysis.

Heavy metals such as Pb, Cd, Cr, Ni, and Zn are commonly associated with military residues, damaged infrastructure, and post-conflict urban contamination pathways [23,24,25,26,27,28,29,30,31,32].

The present study aims to assess the water quality of the Tigris River in post-conflict Mosul, with a particular focus on heavy metal contamination and its implications for drinking and domestic use. Specifically, it (i) quantifies the concentrations and spatial distribution of key heavy metals (Cd, Pb, Cr, Ni, and Zn), (ii) evaluates overall water quality using established indices (CCME-WQI and HPI), and (iii) assesses associated human health risks, while providing spatial mapping of pollution patterns along the river Furthermore, the study identifies contamination hotspots and quantifies their associated health risks. A key novel contribution lies in analyzing the temporal behavior of heavy metals in war-affected areas, enabling the evaluation of whether environmental conditions have deteriorated or shown signs of recovery over time.

Previous studies on the Tigris River in Mosul have primarily focused on general water quality assessments or limited measurements of heavy metals, often without addressing the long-term impacts of armed conflict. Although pre-conflict studies established baseline conditions and some post-conflict investigations reported elevated contamination levels, these efforts were generally constrained by limited spatial coverage, lack of temporal analysis, and absence of human health risk evaluation.

As a result, a comprehensive understanding of the persistence, spatial variability, and toxicological implications of heavy metal contamination in the post-conflict period remains limited. By integrating hotspot identification, spatial–temporal analysis, water quality indices, and human health risk assessment, this study provides a more comprehensive and novel framework for evaluating contamination dynamics in conflict-affected river systems.

## 2. Materials and Methods

### 2.1. Investigation Area

The Tigris River, which flows through the urban area of Mosul, has a total catchment area of approximately 375,000 km^2^ and extends upstream in the northwest to Al Kuba and in the southeast to Hammam alil. In this region, the Tigris River has a total flow length of approximately 214 km, with a width ranging from 50 to 200 m and an average depth of about 50 m. The river gradient is approximately 1:2000, and the average annual discharge ranges between 250 and 400 m^3^/s [33,34]. The hyporheic interstitial zone is composed of two distinct layers: an upper layer of gravel with an average grain size of 32 mm, and a lower layer comprising a mixture of gravel and sand with an average grain size of 13 mm. The gravel morphology is predominantly disk-shaped, followed by flat and spherical forms [34,35,36]. Sampling sites within the catchment area were selected as outlined in Table 1.

**Figure 1:**
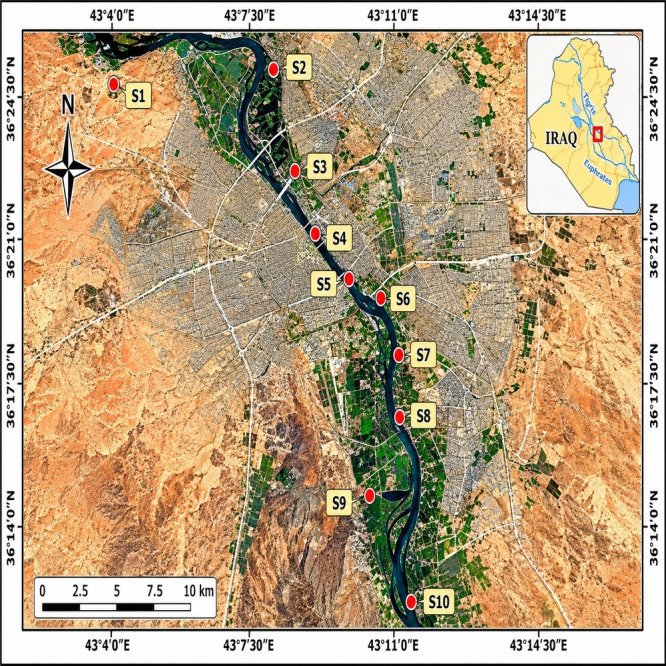
Map of Mosul with highlighted locations of sampling.

The pollution of the Tigris can be divided into a total of four zones (with localised sites in brackets) along its flow length in the urban area. Zone 1 (S1) is characterised as a reference upstream of the confluence with the city and thus as a slightly contaminated water body, as essentially only discharges of agricultural wastewater occur here. The zone is otherwise characterised by recreational areas, which means that the influence of municipal drainage is still low here. In Zone 2 (S2–S4), on the other hand, the Al Khosr valley drains into the Tigris. The river of the same name carries a heavy pollutant load on its course from the north-eastern valleys through this densely populated area and therefore has a high pollutant load [37]. Zone 3 (S5–S7) is characterised by the old town of Mosul, where the military operations to liberate Mosul were concentrated. Finally, Zone 4 (S8–S10) is characterised by an area with intensive agricultural use and residential development outside the direct conflict zone. Details can be found in Figure 1 and Table 1.

### 2.2. Sampling Procedure

A total of 120 water samples were collected from ten predefined locations along the Tigris River within the study area (see Table 1 and Figure 1). At each site, samples were collected in triplicate across four distinct sampling campaigns conducted over a two-year period: series 1 (January–April 2022), series 2 (July–October 2022), series 3 (January–April 2023), and series 4 (July–October 2023). The triplicate samples were analysed separately, and the results are presented as mean values with corresponding standard deviations. All samples were collected in clean, 250 mL polyethylene bottles. Standard protocols were followed to minimise contamination, and water samples were consistently collected from a depth of 0.5 m below the surface.

The temperature of the samples during sampling ranged from 0–45 °C, reflecting seasonal and diurnal variations. Immediately after collection, all samples were stored in a refrigerated container at −4 °C to minimise biological activity and prevent physicochemical changes prior to analysis, in accordance with standard preservation practices. Samples were then transported to the laboratory and analysed for the selected parameters: pH, electric conductivity (EC), % salinity, total dissolved solids (TDS), chemical oxygen demand (COD), ${SO}_{4}^{2-}$, ${PO}_{4}^{3-}$, ${NO}_{3}^{-}$, Pb, Zn, Cd, Cr, and Ni.

The collection and analysis of the samples in the environmental laboratory were carried out according to standard methods [38]. The water flow rate during the period of the study was about 250–300 in series 1 and 2 and about 350–400 m^3^·s^−1^ in series 3 and 4.

Due to the lack of rainwater in 2023, the gates of the Mosul Dam were opened to meet the water requirements for agricultural use in southern Iraq.

Water samples were collected following the [38] standard. During sampling from the riverbank, particular care was taken to ensure that samples were obtained from a representative and well-mixed water layer, avoiding surface films, stagnant zones, and disturbed bottom sediments. Sampling was conducted at approximately 0.5 m below the water surface to ensure consistency and minimise surface-related variability.

### 2.3. Field Analytical Procedures

Field measurements of temperature, pH value, E.C., TDS, and % salinity of water samples were directly analyzed on-site using an Oumefar 5-in-1 digital water quality analyser (Oumefar UPC 886108495111, Oumefar, Shenzhen, China).

### 2.4. Laboratory Analytical Procedures

Phosphate (PO_4_^3−^, in mg/L) was photometrically determined as orthophosphate without prior digestion, following the molybdenum blue method at a wavelength of 690 nm using ammonium molybdate solution. Nitrate (NO_3_^−^, in mg/L) was analysed photometrically at 324 nm after acidification of pre-diluted samples with HCl. Sulfate (SO_4_^2−^, in mg/L) was quantified via precipitation from an acetic acid medium using barium chloride crystals, forming barium sulfate. Chemical oxygen demand (COD, in mg O_2_/L) was determined according to standard methods (APHA; AWWA; WEF) [38].

Heavy metals (Cd, Pb, Cr, Ni, and Zn) were analysed using atomic absorption spectrometry (AAS) with a Phoenix-986 AAS instrument (Phoenix, Duisburg, Germany) following acid digestion.

Absorbance readings were converted to concentrations (µg/L) using calibration curves established according to (APHA; AWWA; WEF) guidelines [38]. Analyses were performed in two stages: (1) total heavy metal concentrations were measured in unfiltered, digested samples; and (2) dissolved metal concentrations were determined in filtered samples (0.25 µm membrane filter) to calculate the heavy metal pollution index (HPI), as described later.

Detection limits (DLs) for the analysed metals were as follows: Cd (0.001 µg/L), Pb (0.005 µg/L), Cr (0.01 µg/L), Ni (0.01 µg/L), and Zn (0.002 µg/L). Method validation was performed through QA/QC procedures, including recovery experiments using spiked samples, which yielded recovery efficiencies ranging from 95% to 105%, indicating high analytical accuracy and precision. Procedural blanks were also included to monitor potential contamination during sample preparation and analysis. All measurements were conducted in triplicate to ensure reproducibility.

Due to the unavailability of certified reference materials, method accuracy was further verified through recovery-based validation. The determination of dissolved heavy metals followed established protocols, as described by USEPA, Leinders et al., and Thakur et al. [39,40,41].

### 2.5. Statistical Analysis Method

Statistical analysis was conducted to evaluate spatial and temporal variations in the measured parameters. Descriptive statistics (mean and standard deviation) were calculated, and differences between datasets were assessed using the *t*-test. A significance level of *p* < 0.05 was applied. All statistical analyses were performed using Microsoft Excel, IBM SPSS Statistics 26 (IBM Corp., Armonk, NY, USA). Spatial distribution maps were generated using ArcGIS 10.8 (ESRI, Redlands, CA, USA).

#### 2.5.1. Assessment of Water Quality and Heavy Metal Consents

The selected indices (CCME-WQI, HPI, and HQ/HI) are widely recognised and frequently applied in water quality and health risk assessments [1,17,18,24].

##### Calculation of the CCME WQI

The Canadian Council of Ministers of the Environment Water Quality Index (CCME-WQI) is a comprehensive tool designed to evaluate water quality relative to established environmental objectives. It offers valuable insights into the effects of water quality on human activities and emphasises critical ecological parameters. This index serves as an effective framework for assessing the condition of the water column, sediments, and aquatic ecosystems, thereby determining the suitability of water for various uses, including human consumption, aquatic life, and wildlife [42,43,44,45,46].

The CWQI equation is computed using three factors as follows:

$$\mathrm{CWQI}\;=100-\{\frac{\sqrt{F1+F2+F3}}{1.732}\}.$$

Factor 1 (F1): Scope: the percentage of the variables that exceed the guideline:

$$F1=\{\frac{Number\;of\;Faild\;Variables}{Total\;Number\;of\;Variables}\}\;\times \;100.$$

Factor 2 (F2): Frequency: this factor represented the percentage of individual tests that do not meet the guidelines (failed tests):

$$F2\;=\;\{\frac{Number\;of\;Faild\;Test}{Total\;Number\;of\;Variables}\}\;\times \;100.$$

Factor 3 (F3): Amplitude: represents the number of readings that exceeded the standards and according to the following steps:

Calculation of excursion: if the values are higher than the values of the standards, the criteria are calculated using the following equation:

$$\mathrm{Excursion}\;=\;\{\frac{Faild\;Value\;i}{Objective\;j}\}\;-\;1.$$

The sum of the standard deviations (nse) and the sum of the readings not meeting the standards are calculated by the sum of the deviations divided by the total sum of the tests:

$$\mathrm{nse}\;=\;\frac{\sum_{i=1}^{n}Excursion}{Number\;of\;Tests}.$$

F3 is then computed from the following equation:

$$F3\;=\;\frac{nse}{0.01\;nse-0.01}.$$

##### Heavy Metal Pollution Index (HPI)

The heavy metal pollution index (HPI) is a rating system designed to reflect the cumulative effect of dissolved heavy metals on water quality, particularly in relation to its suitability for human consumption [47,48,49].

$$\mathrm{HPI}=\sum_{i=1}^{n}\left(Wi\times Qi\right)/\sum_{i=1}^{n}Wi$$

The calculation of weightage of the parameter:

Wi = K/Si.

The quality rating for each of the heavy metal Qi.

$$\mathrm{Qi}=\sum_{i=1}^{n}\frac{Mi-Ii}{Si-Ii}\times 100$$

HPI for each element = (Wi × Qi)/$\sum_{i=1}^{n}Wi$.

Unit weight (Wi): the relative weight assigned to each heavy metal parameter, reflecting its potential impact on water quality. Constant (K): a proportionality constant, typically set to 1 for simplification. Monitoring value (Mi): the observed concentration of the heavy metal in the water sample. Ideal value (Ii): the optimal or desired value of the parameter, often zero for toxic heavy metals. Standard value (Si): the maximum permissible concentration of the parameter, based on guidelines. Number of parameters (n): the total number of heavy metal parameters considered in the analysis.

##### Human Health Risk Assessment

The potential non-carcinogenic health risks associated with heavy metal exposure through drinking water ingestion were assessed using the hazard quotient (HQ) and hazard index (HI), following the guidelines of the United States Environmental Protection Agency [1].

$$\mathrm{HQ}=\frac{ADD}{RfD}$$

where ***HQ*** is the hazard quotient, ***ADD*** is the average daily dose (mg/kg/day), and *RfD* is the reference dose for each metal.

$$\mathrm{ADD}=\frac{C\times IR\times EF\times ED}{BW\times AT}$$

where *C* is the concentration of the metal in water (mg/L), *IR* is the ingestion rate (2 L/day), *EF* is the exposure frequency (365 days/year), *ED* is the exposure duration (30 years), *BW* is the average body weight (70 kg), and *AT* is the averaging time (***ED*** × 365 days).

The cumulative non-carcinogenic risk from multiple heavy metals was estimated using the hazard index (HI):

HI = ∑HQ 

An ***HQ*** or HI value less than 1 indicates no significant risk, while values greater than 1 suggest potential adverse health effects.

## 3. Results and Discussion

### 3.1. Physico-Chemical Characteristics of All Parameters

#### 3.1.1. pH Value

**Figure 2:**
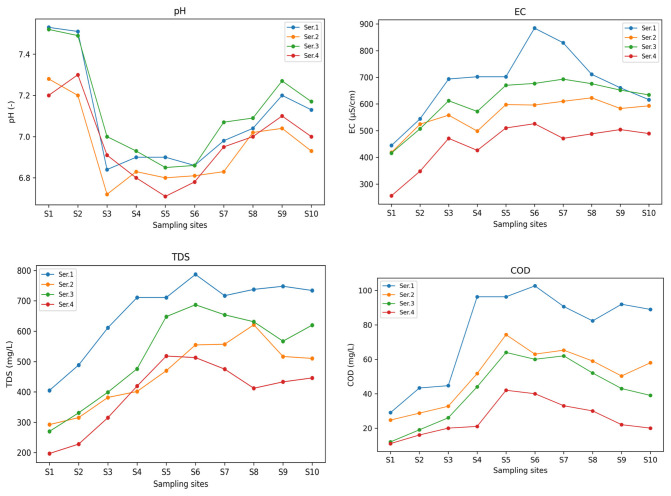

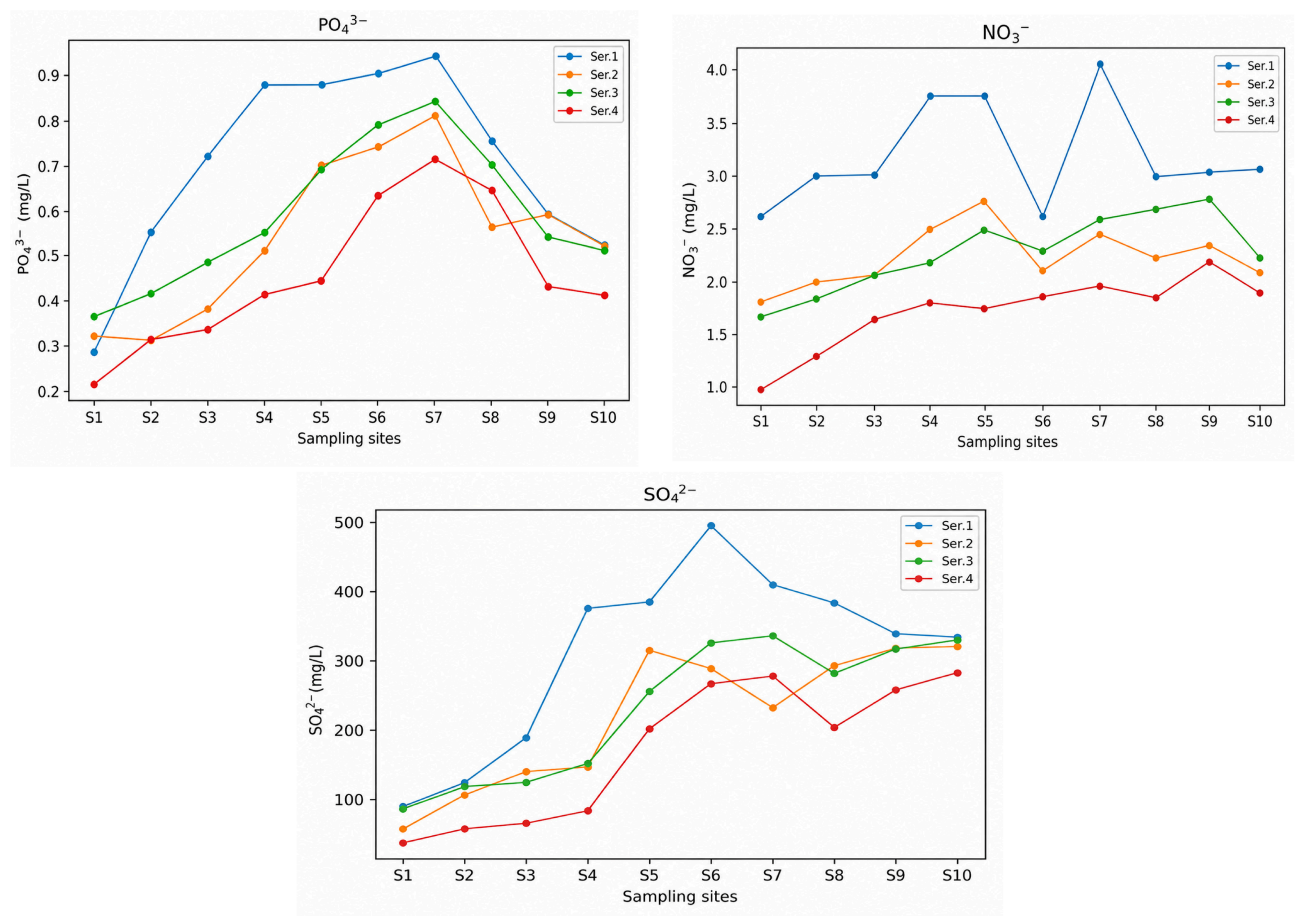
Spatiotemporal dynamics of physicochemical properties in urban river monitoring stations.

The pH values at all sampling sites remained within WHO guideline limits [50]. However, pH decreased along the Tigris River within the urban conflict zone from 7.5 to 6.8 in 2022 and from 7.4 to 6.8 in 2023, likely due to uncontrolled discharges from damaged sewer systems, waste dumping, and contaminated surface runoff. The biodegradation of organic pollutants contributed to partial acidification under oxygen-limited conditions. Seasonal variations were also observed, with lower pH reductions during the rainy season (series 1 and 3) compared to the dry season (series 2 and 4), mainly due to increased river discharge, enhanced turbulence and oxygenation, lower water temperatures, and reduced biodegradation rates (Table 2 and Table 3, Figure 2). Nevertheless, the pH decline remained limited (<0.67 units) because of the buffering effect associated with the carbonate hardness of the river water [5,7].

#### 3.1.2. EC, TDS, and Salinity

The electrical conductivity (EC) of the Tigris River remained below WHO guideline limits at all sites and during all sampling campaigns (2022: 431–740 µS/cm; 2023: 336–602 µS/cm), indicating no restriction for drinking or domestic use [5,50]. However, EC increased progressively along the urban river section, reaching maximum values at S6 before slightly decreasing downstream toward S10. Due to the strong correlation between EC and TDS, similar spatial trends were observed for TDS, which increased from 348.7 to 602.2 mg/L in 2022 and from 233.5 to 583 mg/L in 2023 between S1 and S5, followed by a moderate downstream increase. Salinity values also increased within the urban area, ranging from 0.40–0.70% in 2022 and 0.21–0.55% in 2023, with the lowest values recorded at S1 and the highest at S5 (Table 2 and Table 3, Figure 2). Despite these increases, all measured values remained within WHO permissible limits.

#### 3.1.3. COD

COD values ranged between 26.8–91 mg/L (2022) and 11.5–53 mg/L (2023) at all sites. In detail, starting at the lowest levels at S1 (2022: 11.5 mg/L; 2023: 26.8 mg/L), levels increased over the length of the river, culminated at S5 (91 and 53 mg/L, respectively), and declined smoothly to 73.5 mg/L (2022) and 29.5 mg/L (2023) at S10 due to biodegradation activity.

#### 3.1.4. ${SO}_{4}^{2-}$, ${PO}_{4}^{3-}$, and ${NO}_{3}^{-}$ as Anions

Phosphate, nitrate, and sulphate concentrations followed spatial trends similar to those observed for the other physicochemical parameters. Phosphate concentrations ranged between 0.3–0.9 mg/L in 2022 and 0.3–0.7 mg/L in 2023, exceeding WHO guideline values by approximately 2–2.5 times. Elevated phosphate levels were mainly associated with untreated wastewater infiltration, detergents, fertilisers, industrial discharges, and conflict-related residues [51,52,53]. Concentrations increased markedly after the Al-Mur Valley confluence and along the densely populated urban section, indicating that domestic and faecal wastewater represented the dominant source. Previous studies identified multiple infiltration points discharging nitrate- and phosphate-rich wastewater into the Tigris River within Mosul [54].

Sulphate concentrations ranged from 74.0–336.5 mg/L in 2022 and 62.5–307.0 mg/L in 2023, with some sites exceeding WHO limits by up to 1.4-fold. In addition to municipal wastewater infiltration, elevated sulphate levels were linked to natural sulphur-rich geology, gypsum dissolution, industrial activities, and polluted inflows entering Mosul Lake [5,55,56]. Nitrate concentrations increased moderately along the urban river section from 2.1 and 1.3 mg/L at S1 to 2.7 and 2.5 mg/L at S9 in 2022 and 2023, respectively, mainly due to municipal wastewater infiltration. The relatively limited increase suggests nitrate respiration under partially anoxic conditions along the river flow path (Table 2 and Table 3; Figure 2).

#### 3.1.5. Heavy Metals

Concentrations of Cd, Pb, Cr, and Ni exceeded WHO guideline limits even at the upstream site (S1) and increased further along the urban river section, particularly within the conflict-affected Old City area (S5–S7).

Cadmium concentrations ranged from 4.5–25.9 µg/L in 2022 and 2.6–18.9 µg/L in 2023, with maximum values recorded at S5/S6 before decreasing slightly downstream. WHO limits were exceeded at nearly all sites, reflecting the influence of urban discharges and limited cadmium precipitation under the observed pH conditions.

Lead concentrations reached 23–165 µg/L in 2022 and 12.9–120 µg/L in 2023, exceeding WHO limits at all sites and showing strong spatial association with densely populated and conflict-affected urban areas. Compared with pre-war studies, post-conflict Pb levels remain substantially elevated, likely due to untreated wastewater, industrial and agricultural inputs, and residues from military activities and ammunition [26,56,57,58,59,60,61,62,63,64,65].

Zinc concentrations varied between 146.5–1544.5 µg/L in 2022 and 262.5–1100.5 µg/L in 2023, increasing markedly through the conflict zone but remaining within WHO permissible limits. Elevated Zn levels were associated with municipal and industrial wastewater, agricultural runoff, and war-related soil contamination linked to munitions residues [61,66,67,68,69].

Chromium concentrations exceeded WHO limits at all sites, ranging from 22.2–73.1 µg/L in 2022 and 17.0–66.0 µg/L in 2023. Elevated Cr levels were mainly linked to urban, industrial, agricultural, and combustion-related emissions, in addition to residues from weapons and military infrastructure [70,71,72,73].

**Figure 3:**
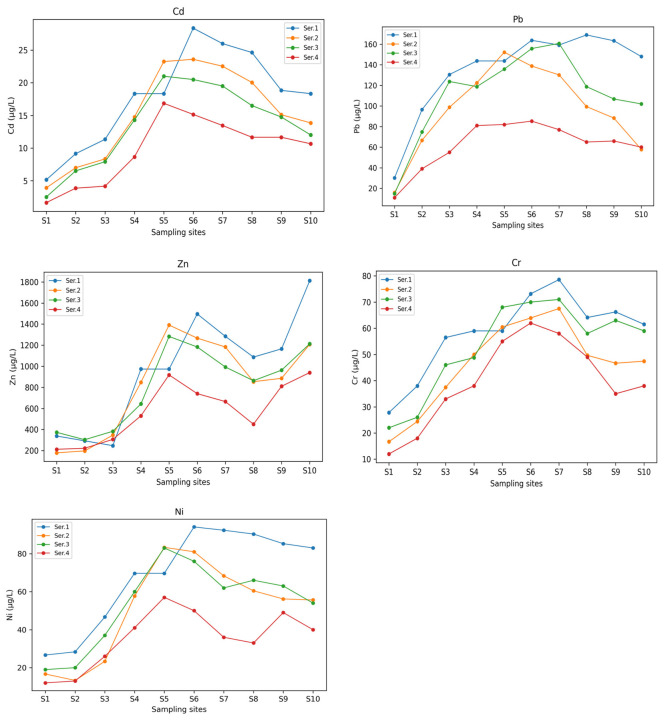
Spatiotemporal dynamics of heavy metal values in urban river monitoring stations.

Nickel concentrations increased from upstream values of 20.8 µg/L (2022) and 15.5 µg/L (2023) to maxima of 90.5 µg/L and 70.0 µg/L at S5, respectively, before declining downstream. WHO limits were exceeded throughout the densely urbanised section, reflecting contributions from surface runoff, industrial emissions, energy infrastructure, and military-related contamination [71,72,73] (see Table 2 and Table 3; Figure 3).

### 3.2. Statistical Analysis

#### 3.2.1. Test Values for Seasonal and Annual Variations

The *t*-test results indicated significant seasonal variation in pH between series 1 and 2 (*p* < 0.05), whereas no significant variation was observed between series 3 and 4 in 2023 (*p* > 0.05). Similarly, no significant annual variation was detected between 2022 and 2023, likely due to the buffering effect associated with the carbonate hardness of the river water [74,75,76,77]. In contrast, EC, TDS, and salinity showed significant seasonal and annual variations (*p* < 0.05), with generally higher values during the wet season (series 1 and 3) compared with the dry season (series 2 and 4) (Figure 2). Similar seasonal increases were observed for phosphate, nitrate, and sulphate concentrations, reflecting the influence of rainfall and increased surface runoff, as also reported by Al-Hamdani [78]. Significant annual variations were detected for phosphate and nitrate [79,80,81], whereas sulphate showed no significant seasonal or annual differences, likely due to the gypsum-rich lithology of the Mosul region and the continuous re-dissolution of sulphate deposits [74,75]. COD values also exhibited significant seasonal and annual variations between the studied series and years, consistent with previous findings [81,82] and the statistical results presented in Table 4 and Table 5.

Seasonal influences were also evident in heavy metal concentrations, with Cd, Pb, Zn, Cr, and Ni generally increasing during the rainy season (winter and spring) and decreasing during the dry season (summer and autumn), resulting in significant seasonal and annual variations (Table 4 and Table 5). Similar seasonal behaviour was observed for EC, TDS, COD, phosphate, and nitrate, mainly due to increased surface runoff and infiltration during rainfall events, which transport salts and pollutants from contaminated soils into the river system [5,6,83,84,85].

Except for sulphate, most parameters showed lower annual fluctuations in 2023 compared with 2022 (Figure 2). This trend was likely related to increased river discharge in 2023 (350–400 m^3^/s versus 250–300 m^3^/s in 2022) following the opening of the upstream dam under low-precipitation conditions, in addition to municipal cleaning activities and debris removal within the conflict-affected areas. Reduced precipitation in 2023 (24.4 mm compared with 40.4 mm in 2022) may also have contributed to lower pollutant transport [86,87,88,89]. Long-term climatic trends in Mosul indicate increasing temperatures and reduced precipitation, which may intensify evaporation, salinity, and future pollutant accumulation under continued environmental stress [89,90]. Reforestation initiatives implemented after the conflict may partially support long-term remediation through the bioaccumulation capacity of vegetation for heavy metals [90].

#### 3.2.2. Comparison of Heavy Metal Concentration with Previous Study

To ensure a reliable comparison, the present results were evaluated against the most comparable pre-conflict study in terms of sampling locations, hydrological conditions, and temporal context, namely the investigation conducted by Aj-Sarraj [54] during 2011–2012. Variations between studies may reflect differences in sampling conditions, river discharge, rainfall, analytical methods, and post-conflict environmental changes, including debris removal and partial recovery processes.

Compared with pre-conflict conditions, Cd and Pb concentrations showed substantial increases across all investigated zones during 2022–2023. Cadmium increased by factors of 4.0 and 2.07 in Zone 1, 6.25 and 4.44 in Zone 3, and 3.69 and 2.57 in Zone 4 for 2022 and 2023, respectively. Similarly, Pb concentrations increased by factors of 4.75 and 3.23 in Zone 1, 6.14 and 4.32 in Zone 3, and 3.17 and 2.66 in Zone 4. Zinc concentrations showed comparatively smaller increases, likely because Zn originates from multiple civilian and industrial sources in addition to conflict-related activities. Detailed concentration values are presented in Table 6.

For quality assurance, the detected heavy metal concentrations were also compared with recent post-conflict studies from Iraq. Aljanabi et al. [91] reported concentrations of 144.8–246.9 µg Pb/L, 14.9–121.4 µg Ni/L, 9.4–19.4 µg Zn/L, and 20.0–71.7 µg Cr/L in the Tigris River within Baghdad, while studies from Mosul during 2020–2021 showed Zn concentrations of 443–1163 µg/L and Pb concentrations of 962–3770 µg/L [61,91].

Comparison with the present study (Zn: 258.4–1509.9 µg/L; Pb: 12.9–165.1 µg/L) indicates that the detected concentrations are generally consistent with previously reported post-conflict contamination levels. Zinc concentrations in Mosul were comparable to those reported in earlier post-war studies, whereas Pb concentrations remained lower than those observed by Kannah et al. [61]. Overall, the comparison confirms a substantial increase in heavy metal contamination relative to pre-conflict conditions, particularly for Cd and Pb, supporting the influence of conflict-related pollution sources on the Tigris River system.

### 3.3. Water Quality Assessment and Analysis

#### 3.3.1. CCME WQI in River’s Water Samples

In this study, the Canadian Council of Ministers of the Environment Water Quality Index (CCME WQI) was employed to comprehensively evaluate the water quality at all sampling sites along the Tigris River within the urban area of Mosul throughout the study period for 2022 and 2023. For these indices, 13 parameters were analysed, namely pH, electrical conductivity (EC), percentage salinity, total dissolved solids (TDS), chemical oxygen demand (COD), sulfate (SO_4_^2−^), phosphate (PO_4_^3−^), nitrate (NO_3_^−^), and the heavy metals (Pb), zinc (Zn), cadmium (Cd), (Cr), and (Ni), which were applied to test the quality of the Tigris River at all sites. The summary of CCME and the values of water samples were presented in Table 1. Regarding the temporal trend, Table 7 shows that S1 resulted in a CCME WQI of ‘good’ with a value of 83.97 (2022) and 90.06 (2023), while S2 was initially classified as ‘fair’ with 74.40 (2022) and 82.65 (2023) due to increasing discharges according to this index, but reached the quality class ‘good’ in the following year. Due to increasing discharges, the water quality deteriorated along the urban watercourse. For example, S3 and S4 fell into the ‘fair’ category in 2022 and 2023, while S5–S7 only fell into the ‘marginal’ category in 2022 (CCME WQI). The water quality of S5 and S7 improved in the following year, so they were categorised as ‘fair’. S8–S10 remained in the ‘fair’ category in both years (CCME WQI). Please also refer to Table 7 for details.

**Table 7:** CCME WQI classification and suitability of all sites for drinking [50].

2022				2023			
Sites	Value	Class	Suitability	Sites	Value	Class	Suitability
**S1**	83.97	4	Good	S1	90.06	4	Good
**S2**	74.40	3	Fair	S2	82.65	4	Good
**S3**	71.53	3	Fair	S3	77.79	3	Fair
**S4**	66.95	3	Fair	S4	70.12	3	Fair
**S5**	62.27	3	Marginal	S5	65.31	3	Fair
**S6**	61.60	2	Marginal	S6	63.85	2	Marginal
**S7**	62.27	3	Marginal	S7	68.59	3	Fair
**S8**	67.89	3	Fair	S8	71.56	3	Fair
**S9**	68.56	3	Fair	S9	71.02	3	Fair
**S10**	68.93	3	Fair	S10	71.56	3	Fair
**Mean**	68.83	3	Fair	Mean	73.25	3	Fair

If all individual sites are combined to form an urban global indicator, the water quality of the Tigris in 2022 and 2023 was in quality class 3 (‘fair’) at 63.83 and 73.25, respectively, with an improvement in water quality already apparent in 2023. This improvement is due in particular to the increased discharge of the river, which dilutes the pollutants, and the cleaning of the riverbanks and debris from the conflict areas. Although no formal studies quantify cleanup activities, local sources and satellite imagery suggest reduced waste accumulation. Similarly, higher river flow during 2023 is also indicated by remote sensing and local hydrological reports.

Nevertheless, the water quality remains poor compared to the pre-war study of 2014 [92], in which the water quality was classified as ‘good’ with a value of 85.8 in class 4.

The CCME WQI values decreased by a factor of 1.18 in 2022 and by a factor of 1.12 in 2023 compared to the pre-war study from 2014 [92]; see also Table 8.

**Table 8:** CCME WQI values, classification, and suitability of the Tigris River during the present study and previous study [92].

Year	Values	Class	Suitability
**2009**	82.848	4	Good
**2010**	88.65	4	Good
**2011**	90.045	4	Good
**2012**	89.852	4	Good
**2013**	80.448	4	Good
**2014**	85.885	4	Good
**2022**	68.83639	3	Fair
**2023**	73.25168	3	Fair

The current water quality of the Tigris River, as assessed using the CCME Water Quality Index (WQI), is categorised as water quality that is generally protected but occasionally threatened or impaired, with conditions sometimes deviating from natural or desirable state values.

#### 3.3.2. Heavy Metal Pollution Index HPI in River Water Samples

Heavy metal pollution index values were calculated using the average values of dissolved heavy metal concentration in the study period 2022 and 2023, according to Table 9. The results show that S1, S2, and S3 could be classified as good and suitable for drinking and domestic use in terms of heavy metal content in 2022 and 2023, while S4–S10 had to be classified as unsuitable for both applications in the same period. An exception is S4, where the water quality improved from ‘unsuitable’ to ‘very poor’ due to an improvement in water quality (see Table 9).

**Table 9:** HPI classification suitability of all sites for drinking [50].

2022			2023		
Sites	HPI	Classification	Sites	HPI	Classification
**S1**	38	good	S1	33	good
**S2**	42	good	S2	37	good
**S3**	48	good	S3	44	good
**S4**	117	unsuitable	S4	87	very poor
**S5**	206	unsuitable	S5	132	unsuitable
**S6**	210	unsuitable	S6	125	unsuitable
**S7**	192	unsuitable	S7	110	unsuitable
**S8**	130	unsuitable	S8	107	unsuitable
**S9**	120	unsuitable	S9	95	unsuitable
**S10**	106	unsuitable	S10	96	unsuitable

The detection of elevated concentrations of (Cd) and (Pb) in the river water is of particular concern due to their high toxicity and persistence in the environment. These heavy metals can enter the human body through multiple pathways, including irrigation of agricultural lands, bioaccumulation in aquatic organisms such as fish, and eventual consumption by local populations [93,94]. Chronic exposure to Cd is associated with kidney dysfunction, skeletal damage, and carcinogenic effects, while Pb is known to impair neurological development, particularly in children, and affect cardiovascular and reproductive systems [95,96]. The presence of these metals at high-risk levels underlines the urgent need for targeted monitoring and remediation strategies to protect both environmental and public health.

#### 3.3.3. Human Health Risk

The calculated hazard quotient (HQ) and hazard index (HI) values for 2022 and 2023 are presented in Table 10.

**Table 10:** Site-specific HQ and HI values for 2022 and 2023.

Sit	Cd	Pb	Cr	Ni	Zn	HI	Cd	Pb	Cr	Ni	Zn	HI
**S1**	51.0	4.4	0.74	0.62	0.05	**56.8**	23.5	2.5	0.57	0.44	0.06	**27.1**
**S2**	91.8	15.6	1.04	0.59	0.05	**109.1**	59.0	11.0	0.73	0.47	0.09	**71.3**
**S3**	111.0	21.9	1.57	1.00	0.05	**135.5**	68.7	17.2	1.32	0.90	0.11	**88.2**
**S4**	187.0	25.4	1.82	1.82	0.30	**216.3**	131.0	19.2	1.45	1.44	0.20	**153.3**
**S5**	283.0	31.5	2.07	2.59	0.51	**319.7**	216.0	21.0	2.05	2.00	0.37	**241.4**
**S6**	294.0	28.9	2.28	2.50	0.46	**328.1**	203.0	23.2	2.20	1.80	0.32	**230.5**
**S7**	275.0	27.6	2.44	2.29	0.41	**307.7**	188.0	22.8	2.15	1.40	0.28	**214.6**
**S8**	252.0	25.6	1.90	2.15	0.32	**281.9**	160.0	17.6	1.78	1.40	0.22	**181.0**
**S9**	192.0	24.0	1.88	2.02	0.34	**220.2**	150.0	16.5	1.63	1.60	0.30	**170.0**
**S10**	182.0	19.6	1.82	1.98	0.50	**205.9**	128.0	12.6	1.62	1.35	0.36	**143.9**

(Cd) was identified as the dominant contributor to overall risk, with exceptionally high HQ values reaching up to 294 in 2022 and remaining critically elevated in 2023 (up to 216). This indicates that Cd is the primary driver of toxicity in the study area. (Pb) also exhibited consistently high HQ values across all sites, further contributing to the cumulative health risk.

The highest hazard index (HI) values were recorded in midstream (conflict area) representing (S4–S7), where HI exceeded 300 in 2022 and remained above 200 in 2023. This pattern reflects pollutant accumulation along the river course and reduced dilution capacity. Although a noticeable decrease in HQ and HI values was observed in 2023, the reduction was not sufficient to bring the risk within acceptable limits (HI < 1).

(Cr) and (Ni) contributed moderate but significant risk levels, particularly in highly impacted sites, while (Zn) showed relatively low HQ values and did not significantly influence the overall risk.

Spatially, the results indicate a clear increase in contamination from upstream to downstream areas, with peak risk levels observed in S4–S7. Temporally, the slight improvement in 2023 suggests partial recovery; however, the persistence of extremely high HI values confirms that the river system remains under severe environmental stress.

Overall, the findings highlight that heavy metal contamination in the Tigris River represents a critical and long-term human health concern, requiring urgent and targeted remediation strategies, particularly in the most affected zones.

#### 3.3.4. Heavy Metals’ Pearson’s Correlation

Pearson’s correlation coefficient for heavy metals indicates a strong correlation between heavy metals in all water sample sites for Pb, Cd, Zn, Cr, and Ni. This suggests a common source of pollution in the sediment. The correlation values indicated that Cd and Pb had very strong correlations with each other and with other metals (Ni, Cr), but the correlation is poorer with Zn, as shown in Table 11.

### 3.4. Geostatic Analysis Arc GIS

#### 3.4.1. The Spatial Distribution Maps of the Tigris River Water Pollution According to CCME WQI

The spatial distribution maps of the Tigris River’s water suitability for drinking and domestic use were created according to the CCME WQI. The distribution of the river’s water suitability for drinking and domestic use was analysed using geostatistical methods in ArcGIS software 10.4 [97]. From the observation of the contamination map, the following highly contaminated sites along the Tigris River were identified:

Approximately 0.702% of the sites, specifically S5 and S6, fall under the “Marginal” category according to the CCME WQI for drinking and domestic use. This is due to their location in the conflict zone (Zone 3).

About 87% of the sites fall under the “Fair” category according to the CCME WQI for drinking and domestic use. These sites are located within the areas surrounding the conflict zone, represented by S3 to S4 and S7 to S10 (Zone 2 and Zone 4).

**Figure 4:**
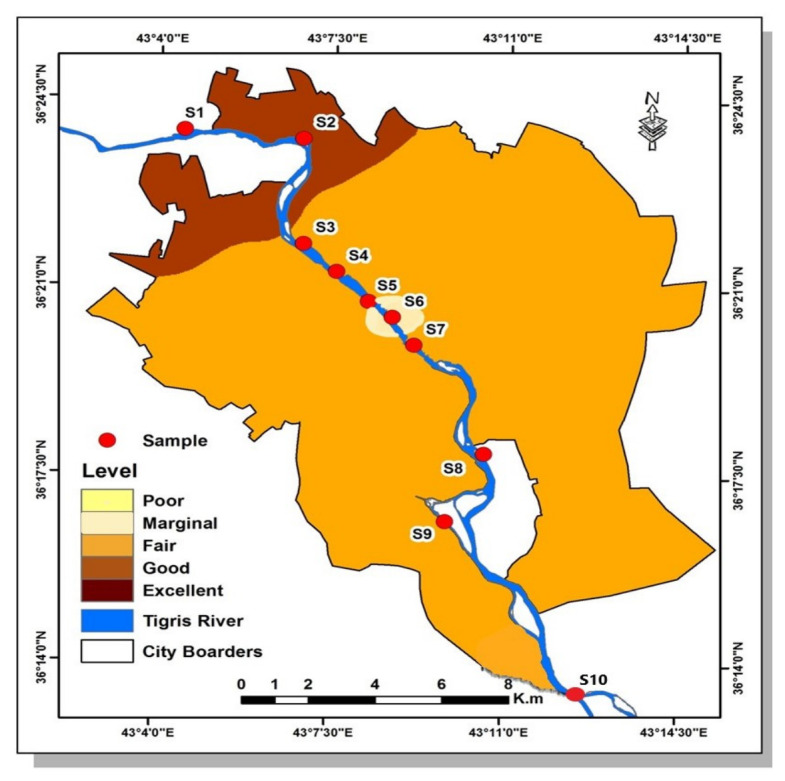
The spatial distribution of Tigris River water suitability for drinking according to the CCME WQI [50].

Around 11.3% of the river’s area falls under the “Good” category according to the CCME WQI for drinking and domestic use. These sites are located at S1 and S2, which serve as reference points before the river enters the city (Zone 1). This area essentially represents contamination from the beginning of agricultural drainage and is characterised by recreational areas at S2, as shown in Figure 4 and Table 12.

#### 3.4.2. The Spatial Distribution Maps of the Tigris River Water Pollution According to Heavy Metal Pollution Index

**Figure 5:**
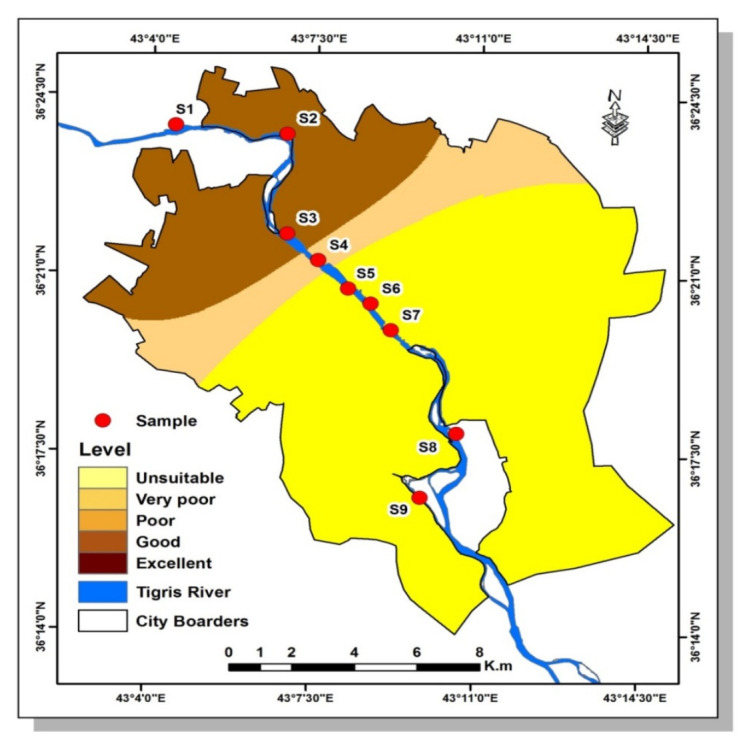
The spatial distribution area of heavy metals according to heavy metal pollution.

The distribution of the heavy metal pollution index (HPI) was analysed using geostatistical methods in ArcGIS software 10.4 [97]. From the contamination map, the following highly contaminated sites along the Tigris River were identified: 65.62% of the sites, classified as “unsuitable” for drinking and domestic use due to their heavy metal content, are located from S4 to S10 (Zone 3, Zone 4). Pollutants enter the river in this area, disperse through the conflict zone, and continue until the river exits the city. In total, 21.84% of the sites, classified as “good” for drinking and domestic use, are located at the river’s entry into the city at S1, S2, and S3 (Zone 1, Zone 2). In total, 12.53% of the sites, classified as “very poor” for drinking and domestic use, are located between S3 and S4 (Zone 2), due to the entry of agricultural drainage from recreational areas. These findings are illustrated in Figure 5 and Table 13.

## 4. Conclusions

This study comprehensively assessed the water quality of the Tigris River in post-conflict Mosul, revealing significant heavy metal contamination and associated human health risks. With the exception of zinc, concentrations of cadmium, lead, chromium, and nickel consistently exceeded international and regional water quality standards at most sites. The overall water quality, as indicated by the Canadian Council of Ministers of the Environment Water Quality Index (CCME WQI), was generally classified as “moderate” (class 3), with slight improvement observed during 2023. However, the Heavy Metal Pollution Index (HPI) categorised the majority of sites (S4–S10) as unsuitable for use, confirming severe pollution conditions, particularly in Zone 3. Importantly, the human health risk assessment revealed alarming results. The calculated hazard quotient (HQ) values for cadmium and lead significantly exceeded the safe threshold (HQ > 1) across all sites, indicating a high likelihood of non-carcinogenic health effects. Cadmium was identified as the dominant contributor to overall toxicity. The cumulative hazard index (HI) values were exceptionally high, exceeding 300 in 2022 and remaining above 200 in 2023 in the most impacted locations. Despite a noticeable reduction in 2023, the risk levels remained far above acceptable limits, confirming the persistence of contamination and limited natural recovery. Spatial analysis demonstrated a clear increase in contamination from upstream to downstream areas, with midstream and downstream zones representing critical hotspots of pollution and health risk. These findings highlight that the environmental impact of conflict extends beyond immediate physical damage, leading to long-term contamination and sustained human health risks.

The novelty of this study lies in the integration of spatial–temporal analysis with water quality indices and human health risk assessment in a post-conflict river system, providing a comprehensive framework for evaluating contamination dynamics. In addition, this study addresses a key scientific question regarding whether post-conflict environments tend to recover or continue to degrade over time, and elucidates the environmental behaviour and persistence of heavy metals released during warfare. The results indicate that, despite partial improvements, contamination remains persistent, suggesting limited natural recovery and ongoing environmental pressure. In addition, the study identifies the most highly contaminated zones along the river, which provides critical guidance for water authorities to avoid raw water abstraction from these areas and to support safer planning of water supply systems.

Crucially, the findings demonstrate the basis for targeted remediation strategies, risk management, and the development of monitoring programs in conflict-affected regions. Armed conflict represents a major contributing factor to the observed heavy metal contamination in the Tigris River. The spatial distribution patterns, strong inter-metal relationships, and persistence of elevated concentrations collectively indicate that war-related activities have significantly influenced current water quality conditions. From a fundamental perspective, the findings advance understanding of the long-term environmental consequences of armed conflict on river systems. From an applied perspective, the results provide a scientific.

Overall, post-conflict environments, such as that of Mosul, pose complex and persistent challenges to river ecosystems, requiring urgent and targeted interventions to mitigate heavy metal contamination and protect public health.

## 5. Recommendations

Continuous monitoring of heavy metals and associated health risks is recommended, particularly in high-risk urban zones. Mitigation measures should focus on wastewater control, remediation of contaminated soils and sediments, and sustainable management strategies. Future research should further investigate long-term ecological and human health impacts, including bioaccumulation and additional exposure pathways, while supporting evidence-based post-conflict environmental policy development.

## Figures and Tables

**Table 1:** Geographic coordinates and descriptions of sampling sites along the Tigris River in Mosul.

Site No.	Location Names	Latitude N	Longitude E	Description
S1	Al Kuba	36.398501	43.074622	Residential area
S2	Alrashidia	36.39606	43.114278	Agricultural area
S3	Third bridge	36.363435	43.114976	Recreation area
S4	Fifth bridge	36.35485	43.12616	Recreation area
S5	Old bridge	36.345667	43.136953	Commercial + residential
S6	Aljumhoria bridge	36.340904	43.145025	Residential area
S7	Fourth bridge	36.332283	43.152415	Residential area
S8	Jarimjah	36.298735	43.176358	Agricultural area
S9	Albosaef	36.277681	43.163921	Agricultural area
S10	Hammam Al aleel	36.160292	43.263505	Residential area

**Table 2:** Characteristics of Tigris River water at all sites during the 2022 tests (series 1 and 2), with corresponding drinking water standard limits [50].

2022		PH	EC µS/cm	TDS mg/L	Sal. %	COD mg/L	${PO}_{4}^{3-}$ mg/L	${NO}_{3}^{-}$ mg/L	${SO}_{4}^{2-}$ mg/L	Cd µg/L	Pb µg/L	Zn µg/L	Cr µg/L	Ni µg/L
WHO Std.		6.5–8.5	1400	1000	1%	100	0.4	50	250	5	10	5000	50	20
S1	Mean	7.5	431.3	348.7	0.4	26.8	0.3	2.2	74.0	4.5	23.0	258.4	22.2	21.7
	±Sd	0.69	89.9	39.9	0.34	5.96	0.114	1.06	55.11	3.82	4.98	91.78	5.78	6.58
S2	Mean	7.4	534.3	402.0	0.5	36.0	0.4	2.5	115.8	8.1	81.6	159.3	31.2	20.8
	±Sd	0.57	83.65	98.49	0.33	6.41	0.104	0.57	29.96	4.58	6.18	100.78	5.48	5.18
S3	Mean	7.0	625.8	496.5	0.6	38.7	0.6	2.5	164.8	9.8	114.6	146.5	47.0	35.0
	±Sd	0.77	107.01	73.25	0.33	6.41	0.104	0.57	29.96	4.8	6.59	137.66	5.8	5.47
S4	Mean	7.0	600.2	556.2	0.7	74.0	0.7	3.1	190.0	16.5	133.1	910.7	54.5	63.7
	±Sd	0.79	151.88	51.88	0.32	4.62	0.074	0.44	22	4.28	5.24	96.46	22.94	7.04
S5	Mean	6.9	706.3	602.2	0.6	91.0	1.0	3.1	344.7	25.0	165.1	1544.5	62.2	90.5
	±Sd	0.9	187.9	92.97	0.4	13.88	0.104	0.57	76.13	7.51	3.36	226.79	14.33	12.02
S6	Mean	6.8	740.2	670.8	0.5	82.8	0.8	2.4	336.5	26.0	151.3	1381.6	68.5	87.5
	±Sd	0.78	173.25	20	0.33	6.41	0.104	0.57	29.96	4.55	5.68	120.26	26.54	7.8
S7	Mean	7.0	719.8	637.0	0.8	78.0	0.9	3.3	277.8	24.3	144.7	1233.7	73.1	80.3
	±Sd	0.72	124	73.25	0.33	6.41	0.104	0.57	30.28	4.73	5.78	111.68	25.06	7.74
S8	Mean	7.0	667.0	679.3	0.6	70.7	0.7	2.6	313.3	22.3	134.2	970.3	56.9	75.4
	±Sd	0.79	110.1	20.1	0.32	3.13	0.064	0.65	29.26	4.75	5.81	112.57	25.24	7.77
S9	Mean	7.1	621.8	632.3	0.5	71.2	0.6	2.7	328.8	17.0	125.8	1025.5	56.4	70.7
	±Sd	0.69	98.16	265.58	0.34	8.94	0.144	0.76	41.54	4.61	5.59	103.67	23.44	7.39
S10	Mean	7.0	604.5	680.2	0.6	73.5	0.5	2.6	327.5	16.1	102.9	1509.9	54.5	69.3
	±Sd	0.55	10.2	10.2	0.31	1.71	0.034	0.38	16.18	4.75	5.81	122.57	25.24	7.77
Zone 1				Zone 2				Zone 3				Zone 4		

**Table 3:** Characteristics of Tigris River water at all sites during the 2023 tests (series 3 and 4), alongside the corresponding drinking water standard limits [50].

2023		PH	EC µS/cm	TDS mg/L	Sal. %	COD mg/L	${PO}_{4}^{3-}$ mg/L	${NO}_{3}^{-}$ mg/L	${SO}_{4}^{2-}$, mg/L	Cd µg/L	Pb µg/L	Zn µg/L	Cr µg/L	Ni µg/L
WHO Std.		6.5–8.5	1400	1000	1%	100	0.4	50	250	5	10	5000	50	20
S1	Mean	7.36	336	233.5	0.213	11.5	0.3	1.3	62.5	2.065	12.9	292.5	17	15.5
	±Sd	0.39	89.60	39.60	0.04	5.66	0.10	0.95	52.33	1.04	2.20	89.00	3.00	3.80
S2	Mean	7.40	428	279.5	0.283	17.5	0.4	1.6	88.5	5.175	56.9	262.5	22	16.5
	±Sd	0.27	83.35	98.19	0.03	6.11	0.09	0.46	27.18	1.80	3.40	98.00	2.70	2.40
S3	Mean	6.96	542	357.0	0.492	23.0	0.4	1.9	95.5	6.025	89.4	344.5	39.5	31.5
	±Sd	0.47	106.71	72.95	0.03	6.11	0.09	0.46	27.18	2.02	3.81	134.88	3.02	2.69
S4	Mean	6.87	499	447.5	0.377	32.5	0.5	2.0	118.0	11.475	99.9	586.5	43.4	50.5
	±Sd	0.49	151.58	51.58	0.02	4.32	0.06	0.33	19.22	1.50	2.46	93.68	20.16	4.26
S5	Mean	6.86	590	583.6	0.545	53.0	0.6	2.1	229.0	18.925	108.9	1100.5	61.5	70
	±Sd	0.60	187.60	92.67	0.10	13.58	0.09	0.46	73.35	4.73	0.58	224.01	11.55	9.24
S6	Mean	6.82	602	600	0.428	50.0	0.7	2.1	296.5	17.825	120.55	961.5	66	63
	±Sd	0.70	172.95	19.70	0.03	6.11	0.09	0.46	27.18	1.77	2.90	117.48	23.76	5.02
S7	Mean	7.01	582	564.5	0.498	47.5	0.8	2.3	307	16.475	118.9	829	64.5	49
	±Sd	0.42	123.70	72.95	0.03	6.11	0.09	0.46	27.18	1.63	2.68	108.58	21.96	4.64
S8	Mean	7.05	582	521.5	0.377	41.0	0.7	2.3	243.0	14.075	91.9	656.5	53.5	49.5
	±Sd	0.49	109.80	19.80	0.02	2.83	0.05	0.54	26.16	1.65	2.71	109.47	22.14	4.67
S9	Mean	7.19	578	500.0	0.392	32.5	0.5	2.5	287.5	13.2	86.4	886.5	49	56
	±Sd	0.39	97.86	265.28	0.04	8.64	0.13	0.65	38.44	1.51	2.49	100.57	20.34	4.29
S10	Mean	7.09	562	533.0	0.435	29.5	0.5	2.1	306.5	11.325	66.3	1076.5	48.5	47
	±Sd	0.25	9.90	9.90	0.01	1.41	0.02	0.27	13.08	1.65	2.71	119.47	22.14	4.67
Zone 1				Zone 2				Zone 3				Zone 4		

**Table 4:** Comparative *t*-test values of all parameters for series 1 vs. series 2 (2022) and series 3 vs. series 4 (2023) to indicate seasonal variation.

*t*-Test Between S1 (Wet) and S2 (Dry) 2022					*t*-Test Between S3 (Wet) and S4 (Dry) 2023			
Parameters	*t*-Test	* DF	*p*	** Status *p* < 0.05	*t*-Test	* DF	** *p*	Status *p* < 0.05
pH	1.745881	18	0.048938	1	1.447636	0.082456	18	0
EC	2.648184	18	0.008176	1	4.165857	0.00029	18	1
TDS	3.868649	18	0.000563	1	2.244035	0.018822	18	1
Salinity%	2.669917	18	0.007808	1	2.244035	0.018822	18	1
COD	2.586038	18	0.009319	1	2.502734	0.011091	18	1
${PO}_{4}^{3-}$	1.826824	18	0.042179	1	1.878644	0.038294	18	1
${NO}_{3}^{-}$	5.394127	18	1.99 × 10^−5^	1	3.51537	0.001235	18	1
${SO}_{4}^{2-}$	1.813528	18	0.021327	1	1.312887	0.102857	18	0
Cd	1.798236	18	0.021757	1	1.474367	0.078829	18	1
Pb	2.008098	18	0.029941	1	2.944581	0.004334	18	1
Zn	1.668797	18	0.025606	1	1.643754	0.05879	18	1
Cr	1.6898	18	0.054156	1	3.256256	0.002192	18	1
Ni	1.484068	18	0.077546	1	2.173495	0.021667	18	1

* DF: degree of freedom, ** *p* < 0.05: significant, *p* > 0.05: non-significant; 0 = not significant, and 1 = significant.

**Table 5:** *t*-test values of annual variation in all parameters between series 1 and series 2 vs. series 3 and series 4.

Parameters	T	*p*	* DF	** Status Value (*p* < 0.05)
**pH**	−0.71769	0.238669	38	0
**EC**	2.425631	0.01007	38	1
**TDS**	2.137765	0.019515	38	1
**Salinity %**	3.525425	0.000561	38	1
**COD**	4.382003	4.48 × 10^−5^	38	1
${PO}_{4}^{3-}$	1.723515	0.046462	38	1
${NO}_{3}^{-}$	4.139127	9.32 × 10^−5^	38	1
${SO}_{4}^{2-}$	1.783523	0.41245	38	0
**Cd**	2.3375	0.01239	38	1
**Pb**	2.235142	0.015682	38	1
**Zn**	1.420289	0.081838	38	1
**Cr**	1.858237	0.035448	38	1
**Ni**	2.035252	0.024422	38	1

* DF: degree of freedom, ** *p* < 0.05: significant, *p* > 0.05: non-significant; 0 = not significant, and 1 = significant.

**Table 6:** Comparison of the heavy metal concentration of the Tigris River in µg/L with previous years; 2013 data taken from [5].

Zone	Element	2013	2022	2023	Factor1	Factor 2
Zone 1	Cd	1	4.0	2.065	4.00	2.07
	Pb	4	19.0	12.9	4.75	3.23
	Zn	469	258.4	292.5	−1.45	−1.38
	Cr	ND	22.2	17	ND	ND
	Ni	ND	21.7	15.5	ND	ND
Zone 3	Cd	4	25.0	17.7	6.25	4.44
	Pb	27	165.1	116.1	6.14	4.32
	Zn	1339	1544.5	963.6	1.15	−1.28
	Cr	ND	62.2	64	ND	ND
	Ni	ND	90.5	60.6	ND	ND
Zone 4	Cd	5	18.5	12.87	3.69	2.57
	Pb	32.5	102.9	86.43	3.17	2.66
	Zn	1961	1509.9	873.17	−1.23	−1.55
	Cr	ND	54.5	50.33	ND	ND
	Ni	ND	69.3	50.83	ND	ND

**Table 11:** Correlation matrix between heavy metals in water samples.

	**C**d	**P**b	**Z**n	**C**r	**N**i
Cd	1				
Pb	0.871326	1			
Zn	0.857698	0.651481	1		
Cr	0.960204	0.920899	0.829294	1	
Ni	0.947679	0.827741	0.884262	0.911433	1

**Table 12:** Spatial distribution area and percentage of the river’s suitability for drinking and domestic use according to the CCWE WQI.

Class	Area (KM2)	Percentage
Marginal	1.417	0.702
Fair	177.49	87.99
Good	22.8	11.3

**Table 13:** Spatial distribution area (square meter) and percentage according to the heavy metal pollution index.

Class	Area (KM2)	Percentage
Good	44.06	21.84
Very poor	25.28	12.53
Unsuitable	132.375	65.62

## Data Availability

The original contributions presented in this study are included in the article. Further inquiries can be directed to the corresponding author.
